# The Impacts of Workplace Environment on Coal Miners’ Emotion and Cognition Depicted in a Mouse Model

**DOI:** 10.3389/fnbeh.2022.896545

**Published:** 2022-06-16

**Authors:** Lei Li, Siwei Wang, Lu Huang, Mei Zhi, Qing Cai, Zihao Fang, Zhenguo Yan, Kaiwen Xi, Dayun Feng

**Affiliations:** ^1^College of Safety Science and Engineering, Xi’an University of Science and Technology, Xi’an, China; ^2^Department of Neurosurgery and Institute for Functional Brain Disorders, Tangdu Hospital, Fourth Military Medical University, Xi’an, China

**Keywords:** coal mine, unsafe behavior, workplace environment, emotion, cognition

## Abstract

Most coal mine accidents are caused by the unsafe behavior of employees. Previous studies have shown that there is a significant connection among the working environment, the psychological state of employees, and unsafe behaviors. However, the internal biological mechanism has not been revealed. To explore the physiological and psychological alterations of coal mine workers and the underlying mechanisms that cause unsafe behaviors, the current study established a novel coal mine environment biological simulation (CEBS) model in mice. This model recreated the underground workplace environment facts in coal mines such as temperature, humidity, and noise, and mice were employed to receive these conditioning stresses according to the 8-h work. Animal behavior tests were performed to evaluate the evolution of the mental state including anxiety and depression, as well as the abilities of learning and memory during the 4-week environmental simulation. CEBS mice showed the adaptation process of anxiety from occurrence to stability in the process of environmental simulation, and also suffered from severe depression compared to the control mice. In addition, impaired spatial memory was also implicated in mice after 4-week CEBS. The behavior results of CEBS mice were consistent with the previous psychological investigation of coal workers. In summary, a novel mouse model was established in this study to depict the occurrence of negative emotions and impaired cognition in coal miners by simulating the underground workplace environment, which provided a basis for further exploring the biological mechanism of miners’ unsafe behavior.

## Introduction

Coal is the main source of China’s energy consumption, and coal mining is also one of the most dangerous industries. According to the China National Coal Safety Supervision Bureau, the death rate per million tons of coal in China fell below 0.1 for the first time in 2018. Although the number of coal accidents has declined in recent years, there are still many coal miners who lose their lives due to coal mine accidents every year (Cao et al., 2019; Liu and Liu, 2020). Numerous accident investigations and the accident caused theory indicated that people’s unsafe behavior is the primary cause of accidents in industrial production (Reason, 1995; Shappell et al., 2007; Yu et al., 2017; Wang et al., 2019). Especially in coal enterprises, 97.67% of coal mine accidents were caused by unsafe behavior (Chen et al., 2013). An increasing number of studies have shown that the occurrence of workers’ unsafe behavior is closely associated with anxiety, depression, and other negative emotions (Leung et al., 2016; Wang et al., 2019; Liang et al., 2022).

Previous studies have shown that coal miners have higher rates of anxiety and depression than other industries (Liu et al., 2014; Deng et al., 2017; Warnier et al., 2020; Xie et al., 2020). Relationships, family environment, income level, work characteristics, physical condition, and many other factors may all affect employees’ mental statuses (Probst and Brubaker, 2001; Adler et al., 2006; Beseler and Stallones, 2010; Deng et al., 2017), and the hostile workplace environment is a marked feature that distinguishes coal miners from other workers (Considine et al., 2017). The high strength and physical labor, high temperature, high humidity, noise, dust, and other harsh workplace conditions led to coal miners’ suffering from a variety of occupational diseases (Liu and Liu, 2020; Li et al., 2021), and seriously causing anxiety and depression symptoms (Liu et al., 2014). These negative emotions are more likely to cause unsafe behavior among coal miners (Yu et al., 2019). In addition, social surveys have found negative effects of environmental factors (temperature, humidity, noise, etc.) in coal mine workplaces on the physiology and psychology of coal miners, while biological effects have been demonstrated in some animal experiments. In a model of cardiac cell injury, high temperature (30 ± 0.5°C) and humidity (90 ± 5%) significantly promoted metabolic disorders in mice (Lan et al., 2022). And mice exposed to aviation noise (peak sound levels of 85 and mean sound level of 72 dBA) had elevated plasma norepinephrine, increased hippocampal neuroinflammation, and cognitive impairment (Munzel et al., 2017; Lin et al., 2022).

However, the mechanism of the influence of the coal mine workplace environment on the psychological state of miners still has not been revealed. Usually, social investigation methods such as questionnaires and scales were used to analyze the effect factors of negative emotions and the relationship between negative emotions and unsafe behavior in coal miners, both of which have subjective, measurement errors and can only be used at a specific time point. With the deepening of the research on unsafe behavior, some scholars try to explore the mechanism of unsafe behavior from a biological perspective. The physiological indicators of employees (e.g., heart rate, heart rate variability, EEG, etc.) are monitored by means of physiological experiments to identify the physiological and emotional characteristics of employees during the evolution of unsafe behaviors (Li et al., 2015, 2018). The method of the physiological experiment is more objective in the results, but it was difficult to analyze the dynamic process and deep physiological changes of emotions affected by the workplace environment. Caballero built an animal model that simulates the coal dust environment of coal to study the effects of coal dust on the physical health of miners (Caballero-Gallardo and Olivero-Verbel, 2016). Although this model cannot accurately simulate the workplace environment of coal miners nor can it describe the dynamic process and characteristics of psychological changes induced by the workplace environment of coal miners, the time validity of the animal model was much higher than that of the previous methods, which hinted that it was possible to build an animal model to study the physiological mechanisms underlying the effects of coal mine workplace environments on mood and behavior. The use of an animal model could reveal the physiological mechanisms underlying unsafe behavior and the impact of the workplace environment on emotion.

Here, we construct a coal mine environment biological simulation (CEBS) model to study the possible impact of coal mine workplace environment on psychology according to the real coal mine workplace environment factors (temperature, humidity, light, noise, etc.) and apply animal behavioral tests to assess how emotions change in response to coal mining environments. This study will attempt to fill the gap in the current field by providing new ideas for revealing the biological mechanism of the psychological impact of the coal mining environment on miners and for pre-identification and medical intervention of unsafe behavior.

## Materials and Methods

### Animal Preparation

Male adult C57BL/6 mice (*n* = 44, 8–12 weeks old, 25.0 ± 2.0 g) were obtained from the Experimental Animal Center of the Fourth Military Medical University. Animals were housed with free bread, food, and water on a 12-h light-dark cycle before the formal experiment in accordance with the National Institutes of Health guide for the care and use of laboratory animals (NIH Publications No. 8023, revised 1,978). All the experimental procedures were approved by the Animal Use and Care Committee of the University.

### Coal Mine Environment Biological Simulation Model

**FIGURE 1:**
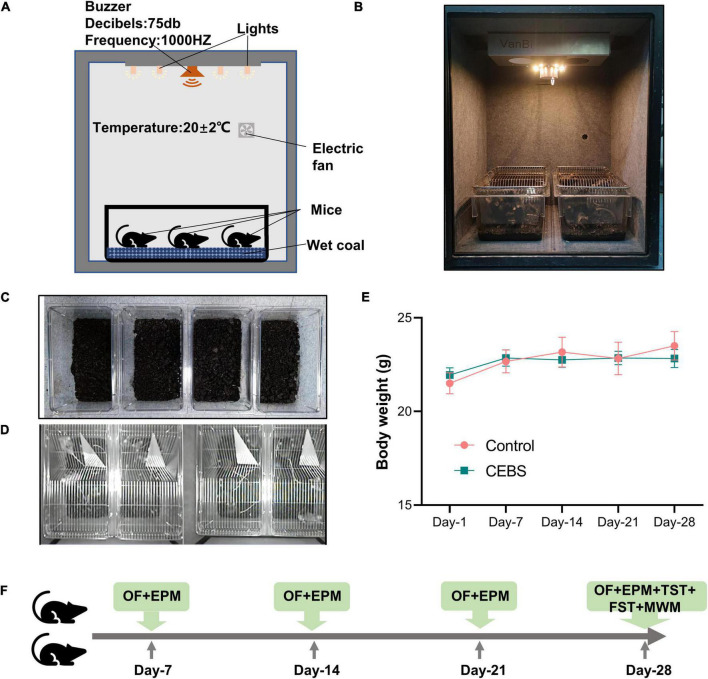
Diagram of the coal mine environment simulator and the overall experimental workflow. The simulation box was used to construct the CEBS model **(A,B)**; the coal ground **(C)**; video screenshot of the modeling process **(D)**; mice were weighed weekly **(E)**. The overall modeling and experimental procedures of this study **(F)**. On modeling days 7, 14, 21, and 28, EPM and OF tests were used to detect anxiety-like behavior. TST and FST were used to detect depression-like behavior on day 28, followed by a 5-day Morris water maze test on the modeling day 29 to detect spatial memory and learning ability. EPM, elevated plus maze; OFT, open field test; FST, forced swimming test; TST, tail suspension test; MWM, Morris water maze. Data are mean ± SEM from *n* = 6 mice (Control), *n* = 20 mice (CEBS: Days 1, 7, 14, and 21), and *n* = 14 mice (CEBS: Day 28) **(E)**.

A special device was built to simulate the workplace environment under the coal mine (Figures 1A, B). The CEBS model device was a rectangular parallelepiped [50 cm (L) * 40 cm (W) * 50 cm (H)]. The white light and buzzer on the top of the box were designed to simulate the light and noise of underground coal mine. The finely divided coal cinder with a thickness of 2–3 cm in the cages [15 cm (L) * 30 cm (W) * 15 cm (H)] was placed as bedding to simulate the ground conditions of the mine. Meanwhile, about 50 ml of pure water was added to the coal cinder (Figure 1C) to keep a moist environment. The modeling processes were implemented as follows: the mice entered the simulated environment (Figure 1D), at 9 a.m. (20 ± 2°C) with exposure to continuous light and noise (1,000 Hz, 75 dB), and then returned to the breeding cage at 5 p.m. There was no food and water supply during the working period. The number of mice in each working and breeding cage was 5. The simulated stimulation time is carried out according to the coal miners’ 8-h daily and 6-day weekly work schedules. Due to the loss of water in the litter during the experiment, about 40 ml of pure water needs to be added every day to maintain the humidity. The simulation experiment lasted for 4 weeks. Toxic gases such as CO, H_2_S, SO_2_, and methane have not been added to the current model because they are present at very low levels in coal mines that meet safety standards (methane: 0–1%, CO: 0–0.0024%, H_2_S: 0–0.005%, SO_2_: 0–0.00025%). The temperature, noise, humidity, and light parameters are set in accordance with the standards of the Coal Mine Safety Regulations (mining face, air temperature: 26°C, noise: 85 dB). The body weight recorded every week indicated that the CEBS modeling method had no damage to the physical signs of mice (Figure 1E).

### Behavioral Tests

The behaviors of depression, anxiety, and learning and memory were measured in CEBS mice (Figure 1F). The elevated plus maze (EPM) and open field test (OFT) were conducted to detect anxiety-like behavior on the seventh day of every week. To reduce the damage to mice, the tail suspension test (TST) and forced swimming test (FST) were only conducted in the last week to detect depression-like behavior. Morris water maze (MWM) test was used to detect the changes in the learning and spatial memory abilities. All the experimental records and index analysis were performed using the behavioral automatic recording system (Noldus EthoVision XT).

#### Elevated Plus Maze Test

**FIGURE 2:**
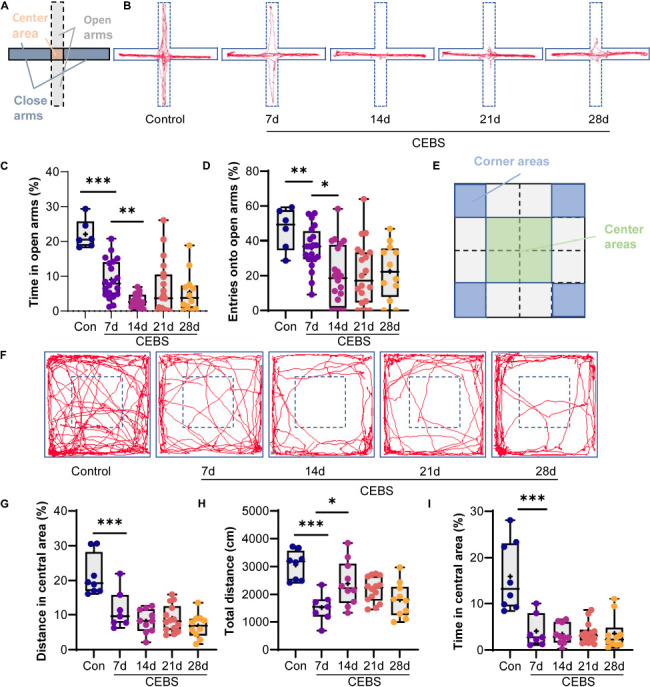
EPM and OF tests showed that the anxiety-like behavior of the CEBS mice had dynamic change. Schematic diagram of EPM **(A)** and OFT **(E)**. Different groups of EPM test motion trajectory diagrams **(B)**. Time in open arms **(C)** and entry onto open arm times **(D)** in the EPM. Different groups of OF test motion trajectory diagram **(F)**. The distance **(G)** and time **(I)** in the center area of the EPM test, the total distance **(H)** in the OFT. Data are presented as mean ± SEM or median (Interquartile range, IQR) from *n* = 6–8 mice/control group, *n* = 20 mice/CEBS group **(C,D)**, and *n* = 7–15 mice/CEBS group **(G–I)**, *P < 0.05, **P < 0.01, and ***P < 0.001.

The elevated plus maze (Figure 2A) consisted of two relatively open arms [30 cm (L) × 30 cm (W)], two relatively closed arms [30 cm (L) × 30 cm (W) × 15 cm (H)], and a central region [5 cm (L) × 5 cm (W)]. The maze was 50 cm above the ground. The EPM test was used to measure the anxiety state of mice by the conflict between the exploration behavior of the new environment and the fear when hanging on the open arms. The detailed procedures are referred to in our previous report (Feng et al., 2019). Briefly, the mice were moved to the room 3 h before the experiment to familiarize themselves with the environment and reduce the influence of the experimental environment on the mice. At the beginning of the experiment, mice were placed from the central region to the open arm, and the number of mice entering the open arm and the closed arm, and the time spent in these two regions were counted for 5 min. Before the next experiment, the feces and urine should be cleaned and the odor wiped with 75% alcohol.

#### Open Field Test

The open-field device was a box [50 cm (L) × 50 cm (W) × 50 cm (H)] (Figure 2E). The bottom of the box was divided equally into 16 small square areas in the behavior analysis software. The four central areas were defined as the center area. The anxious state implicated the conflicts between the curious and fearful psychology of the mice in the unfamiliar open environment. Mice were moved into the lab room for 3 h to familiarize themselves with the experimental environment. Briefly, the mice were placed in the central area and allowed to explore freely for 5 min (Chiba et al., 2012). Feces and urine were eliminated to provide a clean environment for subsequent experiments.

#### Forced Swimming Test

**FIGURE 3:**
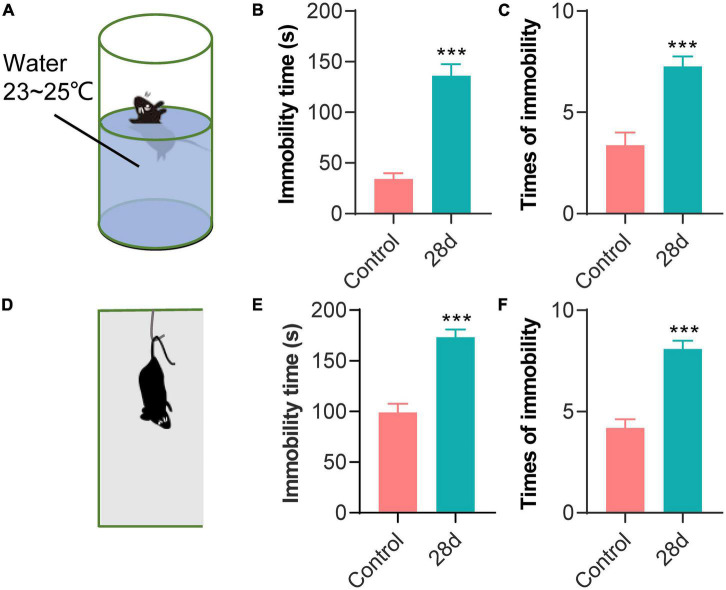
FST and TST showed that the depressive-like behavior of the CEBS mice was obvious. Schematic diagram of FST **(A)** and TST **(D)**. Time **(B)** and times **(C)** of immobility in FST; time **(E)** and times **(F)** of immobility in TST. Data are presented as mean ± SEM or median (Interquartile range, IQR) from *n* = 11–12 mice/group **(B,C,E,F)**, ***P < 0.001.

The FST device is a transparent plastic cylinder [46 cm (H) × 20 cm (D)] (Figure 3A). Mice were placed individually into the cylinders filled to a depth of 20 cm with water (23–25°C) for 6 min (Molendijk and de Kloet, 2019). FST was designed to drive animals into a behavioral despair state by creating a stressful and restricted water environment. The mice that succumbed showed an immobility state, which was defined as the absence of any behavior other than the mouse actively moving upwards to avoid being submerged in the water. The time and number of times the mice being in the immobility state in the last minutes were counted, due to the fact that the mice would struggle violently in the first 2 min.

#### Tail Suspension Test

The TST device is a box [30 cm (L) × 20 cm (W) × 15 cm (H)] with a hook fixed on the top for hanging the mouse tail (Figure 3D). Mice were suspended by the tail using adhesive tape affixed 1/3 from the origin of the tail tip for 6 min, according to the previous report (Zadeh-Ardabili et al., 2019). The suspended mouse went into an immobility state due to despair. The immobility state was defined as a state of small movements of only the forelegs but not the hind limbs, and the swing caused by inertia could be judged as immobile. The time and times of immobility were evaluated in the last 4 min.

#### Morris Water Maze Test

**FIGURE 4:**
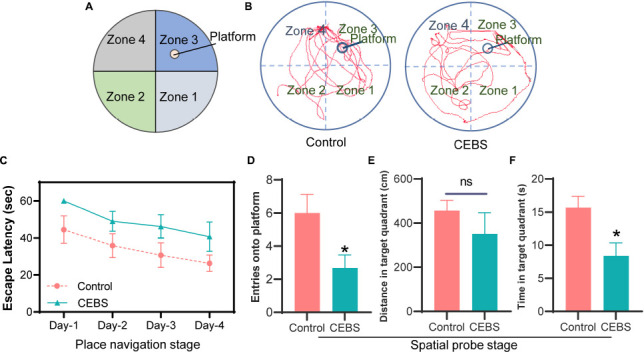
MWM test showed that the spatial memory of CEBS mice was impaired. Quadrant division of MWM **(A)**. The learning ability change was evaluated in the place navigation stage **(B)**. Trajectory diagram of control group and CEBS group in the spatial probe stage **(B)**. Entries onto platform **(D)**, Distance **(E)** and time **(F)** in the target quadrant were used to evaluate the spatial memory ability. Data are presented as mean ± SEM or median (Interquartile range, IQR) from *n* = 6 mice/group **(C–F)**, ns *P* > 0.05, *P < 0.05.

The MWM was performed in a circular pool [160 cm (diameter)] containing a circular platform [15 cm (diameter)] (Figure 4A), and white opaque water (20 ± 2°C) was added to the circular pool to a height of approximately 1 cm above the platform. Four quadrants were artificially divided, and the platform area was marked in the maze in the analysis software. The Morris water maze was performed as previously described (Voet et al., 2018). The MWM test procedure was divided into two stages. The learning ability was examined in a 4-day place navigation test. Mice were individually placed into the pool from the fixed entry points of the four quadrants facing the pool wall, and the time they found the hidden platform was recorded (Escape latency). If the mice did not find the hidden platform after 60 s of traveling, they should be guided to the platform (the escape latency was defined as 60 s). The mice were then taken out and wiped after observing the surrounding environment for 15 s. Each quadrant was trained 15 min apart. The spatial probe test was conducted to test the spatial memory ability on the fifth day. The platform was taken out and mice were dropped from the opposite quadrant of the platform into the pool. The activity of mice in the target quadrant was recorded within 60 s.

### Statistical Analysis

The Student’s *t*-test or one-way ANOVA test were used to test the significance between different groups for data that conforming to the normal distribution, and data were presented as the mean ± standard error of the mean (SEM). The Kruskal–Wallis *H*-test was used to analyze the significance for data that does not conform to the normal distribution, and the data were presented as the median (Interquartile range, IQR). All statistical analyses were performed with the SPSS 26.0 software.

## Results

### Coal Mine Simulated Workplace Environment-Induced Dynamic Change of Anxiety in Mice

CEBS mice were evaluated for anxiety-like behavior using EPM on 7, 14, 21, and 28 days, respectively (Table 1 and Figure 2B). The Kruskal–Wallis *H*-test showed the percentage of time onto open arms in the CEBS group was of significant difference compared to the control group (Figure 2C). Time onto open arms was analyzed by pairwise comparison test: The time decreased continuously from 7 to 14 days compared with the control group (*P* < 0.001, CEBS-7 days group vs. control group; *P* < 0.01, CEBS-14 days group vs. CEBS-7 days group), but remained stable thereafter (*P* > 0.05, CEBS-21 days group and CEBS-28 days vs. CEBS-14 days group). The Kruskal–Wallis *H*-test showed coal environmental simulation had a significant effect on the percentage of entries onto open arms (Figure 2D). Entries onto open arms also decrease continuously from 7 to 14 days and then reached to peak after 21 days (pairwise comparison test, *P* < 0.01, CEBS-7 days group vs. control group; *P* < 0.05, CEBS-14 days group vs. CEBS-7 days group; *P* > 0.05, CEBS-21 days group and CEBS-28 days vs. CEBS-14 days group).

**TABLE 1:** Basic data of EPM and OFT.

Variables	Control	7 Days	14 Days	21 Days	28 Days	F	Sig.
**EPM**							
Time onto open arms	20.53 (7.07)	7.91 (9.31)	1.47 (4.85)	3.73 (10.46)	3.74 (6.61)	26.32	0.000
Entries onto open arms	49.37 (23.03)	36.84 (16.15)	18.62 (36.21)	20.00 (28.07)	22.25 (27.86)	18.07	0.000
**OFT**							
Distance in the center area	19.25 (11.22)	9.60 (8.11)	8.28 (6.24)	8.21 (6.81)	6.89 (4.89)	20.99	0.000
Time in center area	13.26 (13.75)	2.75 (6.62)	2.84 (4.45)	3.13 (2.34)	2.26 (3.68)	18.31	0.001
Total distance	3060.88 ± 183.90	1508 ± 198.32	2392.98 ± 256.71	2164.01 ± 121.64	1787.99 ± 198.14	7.96	0.0000

The OF test was also, respectively, performed to evaluate the anxiety of mice on 7, 14, 21, and 28 days (Table 1 and Figure 2F). The Kruskal–Wallis *H*-test showed a significant difference when the distance in the center area (Figure 2G) and time in the center area (Figure 2I) were compared between the CEBS group and control group. The percentage of distance in the center area (Figure 2G) was compared by pairwise comparison: the distance in center area significantly decreased in the first week and remained stable after (*P* < 0.001, CEBS-7 days group vs. control group; *P* > 0.05, CEBS-14 days group, CEBS-21 days group, and CEBS-28 days group vs. CEBS-7 days group), and time in the center area (Figure 2I) showed the same change (pairwise comparison, *P* < 0.001, CEBS-7 days group vs. control group; *P* > 0.05, CEBS-14 days group, CEBS-21 days group, and CEBS-28 days group vs. CEBS-7 days group). One-way ANOVA test showed a significant difference when the total distance (Figure 2H) was compared. Bonferroni’s *post hoc* test revealed that the total distance significantly decreased in the first week (*P* < 0.001, CEBS-7 days group vs. control group), increased from 7 to 14 days (*P* < 0.05, CEBS-14 days group vs. CEBS-7 days group), and then remained stable (*P* > 0.05, CEBS-21 days group and CEBS-28 days group vs. CEBS-14 days group). The EPM test and OF test demonstrated that the anxiety of CEBS mice appeared to dynamically change during the environmental simulation.

### Coal Mine Simulated Workplace Environment-Induced Depression in Mice

FST was used to evaluate depression-like behavior on day-29 of CEBS induction (Table 2 and Figure 3A). The time (Figure 3B, *P* < 0.001) and times (Figure 3C, *P* < 0.001) of immobility were both significantly increased in the CEBS-28 days group compared to the control group.

**TABLE 2:** Basic data of FST and TST.

Variables	Control	28 Days	Sig.
**FST**			
Immobility time	34.36 ± 5.71	128.50 ± 13.00	0.001
Times of immobility	3.36 ± 0.63	7.27 ± 0.49	0.001
**TST**			
Immobility time	98.91 ± 8.88	173.25 ± 7.78	0.001
Times of immobility	4.00 (1.00)	8.00 (2.00)	0.001

The same statistics method and indicators as FST were used in TST analysis to measure depression-like behavior (Table 2 and Figure 3D). The Student’s *t*-test showed immobility time in the CEBS-28 days group was significantly higher than that in the control group (Figure 3E, *P* < 0.001). The Kruskal–Wallis *H*-test showed a significant difference when times of immobility were analyzed (Figure 3F, *P* < 0.001). The results of FST and TFT both indicated that a coal workplace environment would induce depression and poor performance in individuals.

### Coal Mine Simulated Workplace Environment Impaired Spatial Memory Ability of Mice

MWM test was performed after the above emotional behavior tests (Table 3 and Figure 4A).

**TABLE 3:** Basic data of MWM.

Variables	Control	28 days	Sig.
**Place navigation stage**			
Day-1	50.00 (34.75)	60.00 (0.00)	0.059
Day-2	36.50 (30.00)	53.00 (22.00)	0.147
Day-3	29.00 (27.00)	49.00 (28.25)	0.075
Day-4	28.00 (19.50)	44.50 (41.25)	0.172
**Spatial probe stage**			
Entries onto platform	6.00 ± 1.13	2.67 ± 0.80	0.037
Time in target quadrant	15.67 ± 1.71	8.41 ± 1.94	0.019
Distance in target quadrant	27.33 (11.25)	26.06 (15.50)	0.240

The Kruskal–Wallis *H*-test showed the escape latency of the control group and the CEBS group had no significant difference in place navigation stage (Figure 4C, *P* > 0.05). The average escape latency of the CEBS group was comparatively higher than that of the control group, although there was no difference in the statistics. In the spatial probe stage (Figure 4B), both entries onto the platform and time in the target quadrant in the CEBS group were significantly decreased compared to that in the control group (Figure 4D, *P* < 0.05; Figure 4F, *P* < 0.05). Kruskal–Wallis *H*-test showed the distance in the target quadrant was no significant difference between the two groups (Figure 4E, *P* > 0.05). The MWM test indicated that the coal mine workplace environment might also impair the learning and spatial memory abilities of the miners, which may be another cause of unsafe behavior.

## Discussion

Although, lots of theories and methods in other disciplines are applied to it, such as computer science (Wang et al., 2018), physiology (Cheng et al., 2013; Lee et al., 2017; Li et al., 2018), and management (Paul and Maiti, 2007), the individual physiological mechanism of coal miners’ unsafe behavior affected by workplace environment has not been deeply studied. Due to the difficulty of carrying out paradigm behavior detection with individual differences in the population, it is a perspective to observe the effect of a simulated coal mine workplace environment on the cognitive behaviors of individuals in animal models. This study established a mouse model that was more consistent with the actual workplace environment of a coal mine on the basis of population investigation and animal model research implicating unsafe behavior reported previously. Behavior experiments were conducted to analyze the impact of the workplace environment on the negative emotions and learning and spatial memory abilities that may lead to unsafe behavior in coal miners. The purpose of this study is to explore the relevant biological mechanisms of the impact of the workplace coal mining environment on the unsafe behaviors of coal miners.

Compared with previous surveys on unsafe behavior or mental health of coal miners, using an animal model to reflect the emotional stimulation of the work environment can reduce the influence of other factors in life, so as to improve the pertinence and accuracy of the results. Therefore, we conducted a mid-long term environmental simulation experiment, including the environmental facts of closed, narrow, noisy, and wet characteristics of coal mines, and excluding the factors of the social background, economy, and interpersonal relationships that might affect emotions. Animal behavior experiments showed that CEBS mice exhibited significant anxiety and depression-like behavior after 4 weeks of environmental simulation. This was consistent with previous research on the mental health of coal miners that anxiety and depression are common and more pronounced than in the rest of the population (Considine et al., 2017; Li et al., 2021). However, the CEBS model only reflected the significant influence of the coal mine workplace environment on negative emotions, further indicating that environmental stress is an important factor to induce the cognitive dysfunction related to unsafe behavior.

Notably, CEBS mice showed different levels of anxiety at different times in the environmental simulation process by EPM and OF behavioral experiments. In the first week of exposure to the simulated coal mine environment, the anxiety level of the stressed mice increased significantly and peaked in the second week. Anxiety levels in the mice were somewhat relieved after the third week. The dynamic changes in anxiety levels in CEBS mice at different times after environmental stress indicated that the CEBS mice experienced a process from acute stress to gradual adaptation after exposure to the simulated coal workplace environment. But such anxiety behavior would remain stable for a long time.

Neal pointed out in a study that new employees undergo a stage of adaption to the workplace environment, and this stage is a period when unsafe behavior in new employees may occur frequently (Neal and Griffin, 2004). Neal’s view and other research on the mental health of coal miners demonstrate the feasibility of using the CEBS model to study the impact of the coal workplace environment on emotions. This adaptation phase may be more obvious when new employees are exposed to underground work in the unique environment according to Neal’s research, but the characteristics and patterns of this adaptation process have not been revealed. The CEBS model reflected the changing rules of miners’ emotions in the process of adapting to the workplace environment. Previous studies based on questionnaire surveys and other cross-sectional research methods found it difficult to continuously track and analyze the same individual or the same group. The CEBS model, using animals as the research object, could realize dynamic tracking of time, and analyze the changes of emotions on a time scale under the stimulation of the workplace environment. The change process of anxiety reflected in the experimental results is still of reference significance for analyzing the emotional changes of new employees when beginning their work in coal mines although our research objects are mice rather than actual miners. Therefore, more psychological counseling and appropriate intervention should be carried out for new employees by coal mining enterprises to prevent unsafe behavior in the adaptation stage.

The CEBS model not only reflects the temporal dynamic characteristics of emotion change but also has good temporal validity in other indicators. A study of coal miners using the Neuro Behavioral Test Combination (NCTB) showed that coal miners with a higher working-age scored relatively lower on the memory factor (Zhang et al., 2019). Cognitive functions such as learning and memory are important factors that affect employees’ work behaviors (Neal and Griffin, 2006), especially unsafe behavior (Fang et al., 2016; Shakerian et al., 2019). However, few researchers have reported the impact of the coal mine workplace environment on learning and memory. Here, we conducted a Morris water maze test on CEBS mice, and the results showed that the learning ability of the CEBS mice was not significantly changed, but their spatial memory ability was significantly impaired compared with the normal mice. It indicated that the workplace environment of coal mines would not only affect individual emotions but also impaired memory ability, which might also be an important factor leading to unsafe behaviors. Our CEBS model is helpful for the subsequent in-depth analysis of the mechanism of memory impairment caused by the coal mine environment at the neurobiological level.

This study mainly discussed the influence of the coal mine workplace environment on brain functions such as anxiety, depression, and cognition, but did not analyze the physiological changes caused by the environmental stimulation. At the same time, the age of miners is also an important factor affecting cognitive function, which was not discussed in the present study. These are the limitations of this study and also the direction of our future research.

## Conclusion

This study established a new animal model to investigate the effects of the coal mine workplace environment on emotion and cognition that might lead to unsafe behavior in coal miners. Animal behavior tests showed that the workplace environment of a coal mine had significant effects on anxiety and depression, and also cause impairment of memory ability. The results of this study were discussed with previous studies on negative emotions and unsafe behaviors of coal miners, indicating that the CEBS model revealed good consistency in simulating the effects of the workplace environment on coal miners. Aided by the CEBS model, we will have a deeper understanding of the impact of the coal mine workplace environment on the unsafe behavior of miners, which provides a good tool and foundation for revealing the mechanism of coal mine workplace environment on unsafe behavior from a biological perspective, and for providing new ways to further reduce unsafe behavior of the coal miners and reduce coal mine accidents.

## Data Availability Statement

The original contributions presented in the study are included in the article/supplementary material, further inquiries can be directed to the corresponding author/s.

## Ethics Statement

The animal study was reviewed and approved by the Ethics Committee of Tangdu Hospital, Fourth Military Medical University.

## Author Contributions

LL: conceptualization, supervision, funding acquisition, and writing—original draft preparation. SW: data curation and formal analysis. LH: investigation and software. MZ: visualization. QC: supervision. ZY: resources. ZF: software. KX: validation. DF: conceptualization, methodology, funding acquisition, and writing—reviewing and editing. All authors contributed to the article and approved the submitted version.

## Conflict of Interest

The authors declare that the research was conducted in the absence of any commercial or financial relationships that could be construed as a potential conflict of interest.

## Publisher’s Note

All claims expressed in this article are solely those of the authors and do not necessarily represent those of their affiliated organizations, or those of the publisher, the editors and the reviewers. Any product that may be evaluated in this article, or claim that may be made by its manufacturer, is not guaranteed or endorsed by the publisher.
